# Effects of *Taenia Pisiformis* Infection and Obesity on Clinical Parameters, Organometry and Fat Distribution in Male Rabbits

**DOI:** 10.3390/pathogens9110861

**Published:** 2020-10-22

**Authors:** David Arias-Hernández, Sara García-Jiménez, Rosa Domínguez-Roldan, Clara Murcia-Mejía, Armida Báez-Saldaña, Claudia Hallal-Calleros, Ivan Flores-Pérez

**Affiliations:** 1 Facultad de Ciencias Agropecuarias, Universidad Autónoma del Estado de Morelos; Cuernavaca 62209, Mexico; dariasher@hotmail.com (D.A.-H.); rosa.dguez88@gmail.com (R.D.-R.); 2 Facultad de Farmacia, Universidad Autónoma del Estado de Morelos, Cuernavaca 62209, Mexico; saragarcia@uaem.mx; 3 Facultad de Medicina Veterinaria y Zootecnia, Universidad Nacional Autónoma de México, Mexico City 04510, Mexico; murcia@unam.mx; 4 Instituto de Investigaciones Biomédicas, Universidad Nacional Autónoma de México, Mexico City 04510, Mexico; armida@biomedicas.unam.mx

**Keywords:** cortisol, comorbidity, fat redistribution, parasites

## Abstract

*Taenia pisiformis* infection causes important economic loss in farms. It is suggested that obesity has a major impact on infection and reproduction. We addressed the impact of *T. pisiformis* infection in normal and obese rabbits to evaluate its effect on parameters important in behavior and reproduction. *T. pisiformis* infection in obese rabbits decreased body weight. In the obese-infected rabbits, eosinophils and heterophiles were increased 23% by the infection (*P* ≤ 0.05). *T. pisiformis* decreased cholesterol by 13% in normal weight infected rabbits and 10% in obese group (*P* ≤ 0.05), while triglyceride and VLDL were increased by 23% and 45% in the non-infected obese group (*P* ≤ 0.05). The infection increased serum cortisol levels only in normal weight rabbits (*P* ≤ 0.05). Liver weight was 20% higher in obese and obese-infected rabbits (*P* ≤ 0.05). Testicular weight in obese-infected was 46% higher than normal weight (*P* ≤ 0.0001) and 20% more than the obese-non-infected (*P* ≤ 0.0001). Furthermore, the infection reduced the weight of submandibular glands in infected and obese-infected rabbits (*P* ≤ 0.05), body fat increased 10% in the obese-infected than in the obese, and infected group was 35% over the normal weight non-infected (*P* ≤ 0.01). Our results show that *T. pisiformis* alters metabolic characteristics in rabbits, which can impact on the production and welfare of animals.

## 1. Introduction

Parasitic diseases affect a third of the world population; both humans and animals are parasitized at some stage of their lives [1]. The metacestode of *Taenia pisiformis* (*T. pisiformis*) is a very frequent parasite in farm rabbits and in pets, being very frequently found in lactating and pregnant wild does [2]. It has been documented that infection in rabbits reduces litter size by 50%, with an increase in serum progesterone levels [3]; the adult stage of *T. pisiformis* causes behavior modifications in experimentally infected hamsters [4] and the metacestode in domestic rabbits [5]. Obesity has been identified in rabbits, dogs, cats, pigs, and other animals, which are commonly considered obese when they are 20% over their ideal weight [6]. In rabbits, obesity occurs in farms mainly in breeding does and bucks, in experimental rabbits, and significantly in companion animals. However, to date, the information regarding the alterations of clinical parameters associated with obesity in the domestic rabbit and the obesity-infection comorbidity are poorly understood. Adipose tissue, besides storing fat, secretes hormones, for what it is considered the largest endocrine organ in the body. The amount of adipose tissue depends on factors such as age, physical activity, and genetics [7], but the impact of factors as infection diseases has not been cleared. Parasitism by cestodes in conditions of obesity has been scarcely studied in animals and humans. However, it has been reported that in obese mice infected with the nematode *Nippostrongylus brasilensis* the weight gain decreased and it was associated with a better metabolism of glucose, with a reduction of adipose tissue and hepatic steatosis, and also with a modified pattern of hormone expression related to metabolism. Considering these findings, it was proposed that infection by nematodes can have preventive or therapeutic effects in the development of obesity and its associated metabolic disorders [8]. It has also been documented that infections with parasites in humans can modify the lipid profile [1], and in mice, *Schistosoma mansoni* can reduce serum cholesterol levels by modulating lipid metabolism, thus counteracting the effect of atherogenic diets [9]. In addition, it has been observed that patients infected with the protozoa *Giardia lamblia* present lower levels of total cholesterol and LDL compared to healthy individuals [1]. In the case of cestodes, specifically in male mice infected with *Taenia crassiceps*, it was found an increase in the number of macrophages in the luminal space and in the seminiferous tubules, and morphological abnormalities in somatic cells (Sertoli and Leydig cells) and in germ cells [10]. The increase in visceral adipose tissue is associated with glucose intolerance and insulin resistance, which is directly correlated with the content of triglycerides in the liver [11]. In rabbits fed an obesogenic diet for 12 weeks, an increase in body weight and liver size was observed. They also had a significant increase in visceral fat, having histometric changes in the liver and testicles [12]. In the present study, we determined the effect of the infection with *T. pisiformis* eggs in obese, as opposed to normal weight rabbits; addressing some clinical parameters such as lipid profile, blood cells, hepatic enzymes and serum cortisol levels, in addition to the distribution of adipose tissue, and organometric changes in the size and weight of chinning glands and testicles.

## 2. Results

### 2.1. Body Weight and Weight Gain

**Figure 1:**
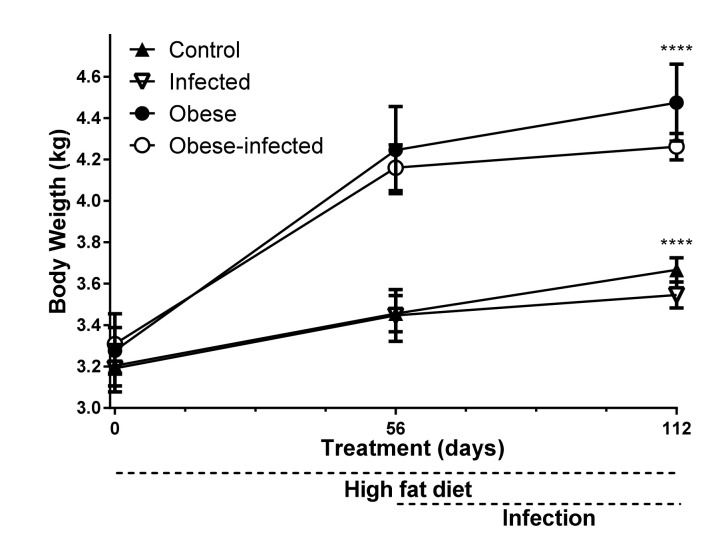
Weight at days zero, 56 (time of infection) and 112 (end of the experiment); the obese and obese-infected groups were fed a high-fat diet during 56 days previous to infection. The infection of the infected and obese-infected groups was performed with 3000 eggs of *T. pisiformis* 56 days after diet. Data show the mean ± SD, **** *P* ≤ 0.0001, ANOVA test, followed by a Tukey´s post hoc test.

Rabbits fed a high fat diet (HFD) for 56 days reached one kilogram more weight than rabbits fed with the maintenance diet, which represents 24% more weight (Figure 1). On day 56 post infection, the weight of the normal weight infected group was lower in relation to the normal weight control group (control vs. infected, 3.67 ± 0.06 vs. 3.55 ± 0.06, *P* ≤ 0.0001). Similarly, the obese-infected group had a lower weight (4.26 ± 0.06) compared to the weight of the obese group without infection (4.47 ± 0.19) (*P* ≤ 0.0001) (Figure 1).

### 2.2. Clinical Parameters

#### 2.2.1. Complete Blood Count, Liver Enzymes and Lipid Profile

Values obtained in the blood count are shown in Table 1, where the changes induced by chronic infection with *T. pisiformis* eggs are observed, compared against the control group. Total red and white blood cells did not show differences between groups; however, eosinophils increased 23% in the infected group (*P* ≤ 0.05) and heterophiles increased by 22% in the obese-infected group (*P* ≤ 0.05); in the lymphocytic, monocytic and basophilic blood cells no differences were observed between the groups. The mean corpuscular hemoglobin (MHC) and mean corpuscular hemoglobin concentration (MCHC) values were also similar in all groups. Serum levels of the liver enzyme alanine aminotransferase (ALT) and aspartate aminotransferase (AST) were unchanged in the comparison between groups. Cholesterol decreased by 13% in the normal weight infected rabbits (*P* ≤ 0.01) and by 10% in obese-infected rabbits (*P* ≤ 0.05) compared to the control group. In the obese group, triglycerides and VLDL increased by 23% (*P* ≤ 0.05) and 45% (*P* ≤ 0.01), respectively, compared against the control group (Table 1), and HDL decrease by 16% in obese-infected compared against the obese group (*P* ≤ 0.01) (Table 1).

#### 2.2.2. Serum Cortisol

**Figure 2:**
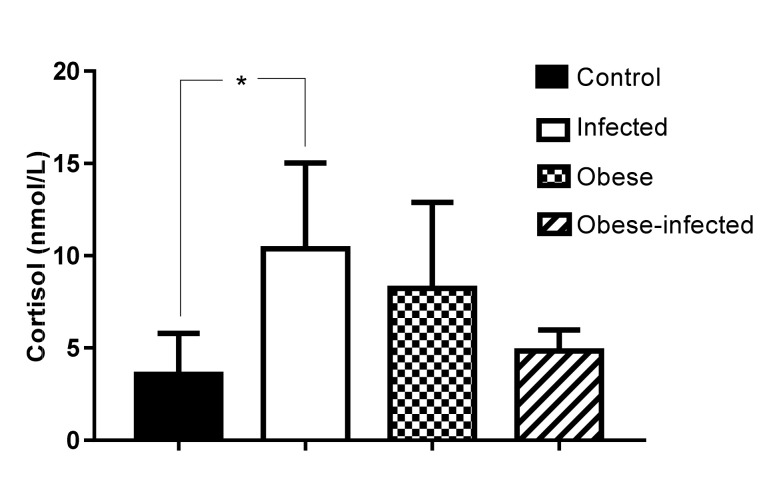
Serum cortisol levels were measured at day 56 post infection in rabbits infected with *T. pisiformis.* Data show mean ± SD, * *P* ≤ 0.05, ANOVA test, followed by a Tukey´s post hoc test.

The levels of serum cortisol in normal weight infected animals showed an increase with respect to the control group (*P* = 0.0132). The comparison between the obese and obese-infected groups showed no difference (Figure 2); although a trend is observed, they were not considered different, since the mean for the obese group was 10.37 ± 4.65 while that of the infected obese group was 4.84 ± 1.12 (*P* = 0.052).

### 2.3. Parasite Load

The analysis at necropsy allowed to observe a higher number of liver granulomas in the infected group, where a total of 25 granulomas were observed in the 85% of the animals. In the obese-infected group, metacestodes were found in 100% of the rabbits, with a range from 4 to 22 metacestodes (Table 2).

### 2.4. Organometric Changes

#### 2.4.1. Weight of Liver, Testicles and Submandibular Glands

**Figure 3:**
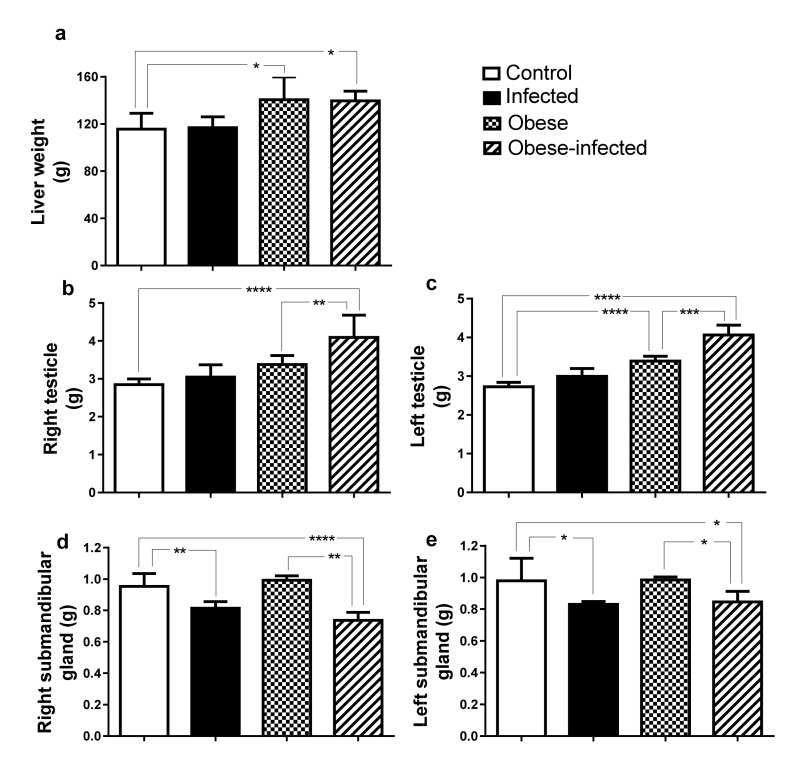
Weight of the liver, testicles and submandibular glands in rabbits infected with *T. pisiformis.* (**a**) Liver, (**b**) Right testicle, (**c**) Left testicle, (**d**) Right and left submandibular weight (**e**). Data show mean ± SD. * *P* ≤ 0.05, ** *P* ≤ 0.01, *** *P* ≤ 0.001, **** *P* ≤ 0.0001, ANOVA test, followed by a Tukey´s post hoc test.

**Figure 4:**
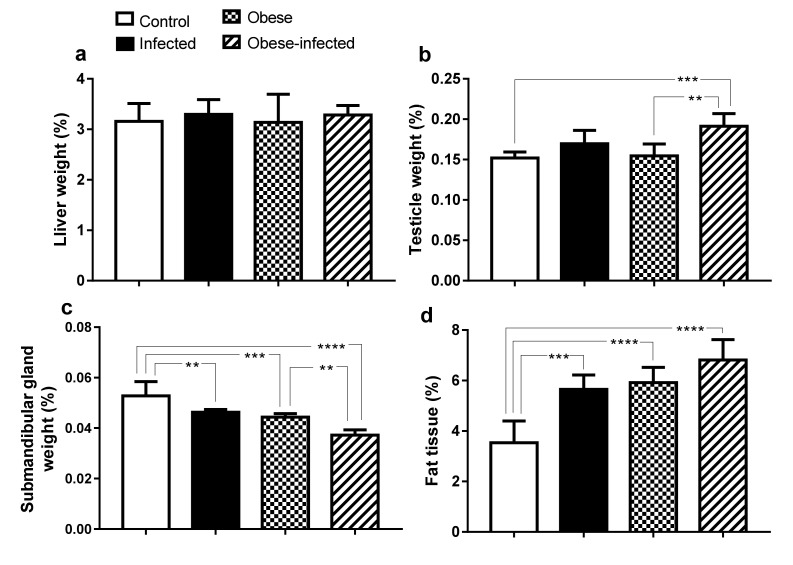
Proportion of the weight of the liver, testes, submandibular glands and adipose tissue in relation to the body weight of rabbits infected with *T. pisiformis.* (**a**) Percentage of the liver weight, (**b**) Percentage of the testicular weight, (**c**) Percentage of the weight of the submandibular glands and (**d**) Percentage of the weight of the adipose tissue. Data show mean ± SD. ** *P* ≤ 0.01, *** *P* ≤ 0.001, **** *P* ≤ 0.0001, ANOVA test, followed by a Tukey´s post hoc test.

Liver weight was 20% higher in the obese and obese-infected groups (*P* ≤ 0.05, Figure 3a). However, the percentage of the liver weight with respect to the body weight of the rabbits was not affected in the different experimental groups (Figure 4a). The testicles of obese-infected rabbits had a 44% increase in weight (right testicle, *P* ≤ 0.0001, Figure 3b) and 49% (left testicle, *P* ≤ 0.0001, Figure 3c). They also had a higher weight in the obese group, resulting in a 21% increase in the right testicle (*P* ≤ 0.01, Figure 3b) and a 20% in the left testicle (*P* ≤ 0.0001, Figure 3c). The comparison of the right testicle versus the left testicle in the same group was performed in all the groups and showed no weight differences. The weight of the submandibular glands was lower in the infected and obese-infected groups compared to the control group (*P* ≤ 0.05). It was also observed that the weight was lower in the glands of the obese-infected versus the obese group (*P* ≤ 0.05) (Figure 3d, e). In the obese-infected group the left submandibular gland is heavier than the right (0.85 ± 0.03 g and 0.74 ± 0.03 g, respectively; *P* = 0.0103).

#### 2.4.2. Percentages of Weight of Liver, Testicles and Submandibular Glands

The percentage of testicular weight was higher in obese-infected rabbits compared to those in the control group (0.19 ± 0.0157 vs. 0.15 ± 0.0073, *P* ≤ 0.001). This increase was also observed when obese rabbits were compared to obese-infected (0.15 ± 0.0144 vs. 0.19 ± 0.0157, *P* ≤ 0.01) (Figure 4b). The percentage of weight of the submandibular glands decreased in the three experimental groups when compared against control; a 11.6% reduction in weight was observed in the infected group, 15.4% less in the obese group (*P* ≤ 0.001) and 28.84% less in obese-infected group (*P* ≤ 0.01). A decrease was also observed when contrasting the obese-infected group against the obese group (*P* ≤ 0.01) (Figure 4c). 

### 2.5. Distribution of Adipose Tissue

**Figure 5:**
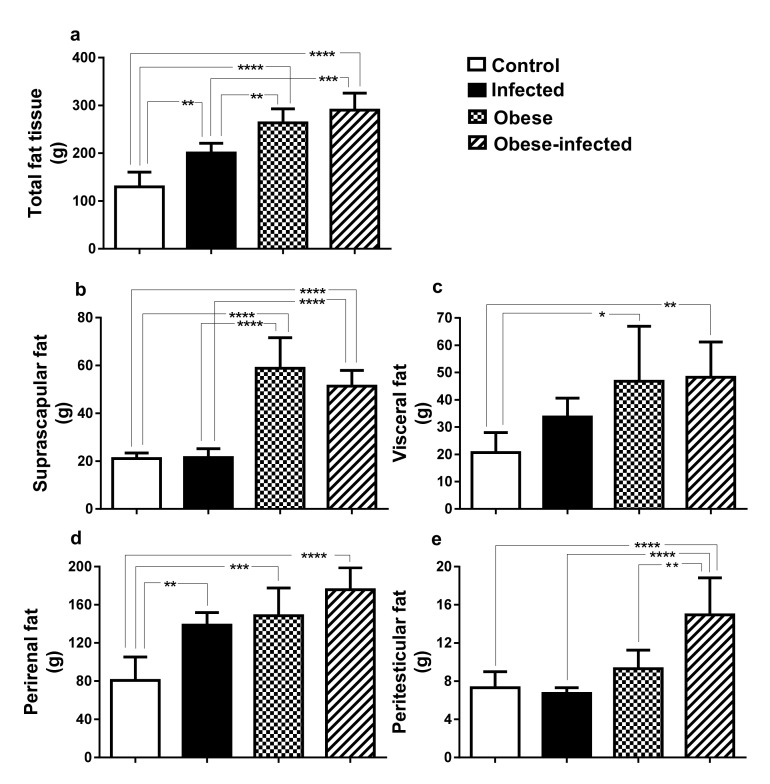
Distribution of body adipose tissue in rabbits infected with *T. pisiformis*. (**a**) weight of total adipose tissue, (**b**) suprascapular, (**c**) visceral, (**d**) perirenal, and (**e**) peritesticular. Data show mean ± SD. * *P* ≤ 0.05, ** *P* ≤ 0.01, *** *P* ≤ 0.001, **** *P* ≤ 0.0001, ANOVA test, followed by a Tukey´s post hoc test.

Total adipose tissue increased in all three experimental groups with respect to control. In infected rabbits, an increase of more than 50% was observed (129.8 ± 30.6 vs. 200.6 ± 20.4 g, *P* ≤ 0.01), whereas in the obese group twice as much adipose tissue was observed (263.5 ± 29.5 g (*P* ≤ 0.0001), and in obese-infected it increased 223% (290.2 ± 35.6 g, *P* ≤ 0.0001). When comparing between groups, the obese (*P* = 0.0073) and the obese-infected (*P* = 0.0002) resulted in a higher amount of adipose tissue in relation to the infected group (Figure 5a). 

We studied the body distribution of adipose tissue in the suprascapular region, the visceral region, the perirenal region, and the peritesticular region. In the suprascapular region adipose tissue increased 1.7 times in the obese group and 1.4 times in the obese-infected compared with the control (*P* ≤ 0.0001) (Figure 5b). Adipose visceral tissue also increased 1.27 times in the obese (*P* = 0.011) and 1.3 times in obese-infected (*P* = 0.007) with respect to control (Figure 5c). The adipose tissue of the perirenal region increased 70% in the infected rabbits in relation to the control group (80.72 ± 24.6 g vs. 138.7 ± 13.2 g, *P* = 0.001); the obese group increased an 80% its adipose tissue content (148.5 ± 29.13 g, *P* = 0.0003) and the obese-infected group (175.7 ± 23.2 g) doubled it (217%, *P* ≤ 0.0001) (Figure 5d). Regarding the peritesticular adipose tissue, the obese-infected group (14.9 ± 3.9 g), increased twice compared against control (7.3 ± 1.7 g vs. 14.9 ± 3.9, *P* ≤ 0.0001), infected (6.7 ± 0.6 g, *P* < 0.0001) or obese groups (9.3 ± 1.9 g, *P* = 0.0025) (Figure 5e).

#### Percentage of Body Adipose Tissue

The percentage of adipose tissue revealed an increase in obese-infected (6.81 ± 0.80) (*P* ≤ 0.0001) and in infected rabbits (5.6 ± 0.57) (*P* ≤ 0.001), compared with 3.53 ± 0.86 in the control group, besides that the obese group also increased its percentage of adipose tissue (5.91 ± 0.60) (*P* ≤ 0.05) (Figure 4d).

### 2.6. Percentage of Re-Distribution of the Adipose Tissue in the Different Anatomical Sites

Table 3 shows changes in the localization of adipose tissue. The comparison of perirenal adipose tissue among the different groups showed a higher percentage in the infected group than in the obese group (69 ± 2% vs. 56 ± 9%, *P* ≤ 0.01). The percentage of suprascapular tissue was highest in the obese group (23 ± 6%) compared to the control group (17 ± 4.3%) (*P* ≤ 0.05), the infected group (11 ± 1.7%) (*P* ≤ 0.001) and the obese-infected (18 ± 1%) (*P* ≤ 0.05). 

There is a decrease in the infected groups (control vs. infected and obese vs. obese-infected). In the percentage of adipose tissue accumulated in the mesentery no difference was observed. Finally, the percentage of peritesticular tissue was higher in the control group (6 ± 2.4%) compared to the infected (3 ± 0.4%) (*P* ≤ 0.05) and obese groups (3 ± 0.5%) (*P* ≤ 0.05).

## 3. Discussion

In the current work, obese rabbits developed a higher number of *T. pisiformis* metacestodes. In animal models and in humans in which the interaction between obesity and infectious diseases has been studied, it has been suggested that obesity increases the susceptibility to infection by bacteria, viruses and protozoa [15], with not previous studies of cestodes infection. The mechanism by which obesity facilitates infection is not well known, however, in some studies in obese mice infected with *Klebsiella pneumoniae* [16] or with *Mycobacterium tuberculosis* [17], a delayed immune response against the pathogen was evidenced. In addition, the phagocytic function was not efficient for the exclusion of the pathogen, which generated a higher mortality.

In our obesity model, infection with *T. pisiformis* decreased body weight at day 56 post infection, affecting mainly obese rabbits, and we also observed a trend to reduce weight gain in normal weight infected rabbits. This data is consistent with the observations in rats infected with the nematode *N. brasilensis*, where the infection induced weight loss [18]. Moreover, in mice subjected to a cafeteria diet, a higher frequency of infection with the influenza virus and higher mortality rate was observed. This observation was attributed to a decrease in chemical mediators such as IFN-α and IFN-β, as well as a reduction in the cytotoxic capacity of NK cells [19]. This finding is consistent with our observation in the obese-infected rabbits, in which the total number of metacestodes was higher and all animals were infected; it is important to note that *T. pisiformis* is a parasite that naturally infects wild and domestic rabbits and hares [2,20]. Therefore, it is of interest to study the mechanisms associated with the susceptibility to infection observed in obese rabbits, especially the role of cytokines in this comorbidity.

Parasitic behavior in an obese host remains unknown [15]. Parasites such as *Toxoplasma gondii* induces a reduction in mortality, in parasitemia, and in cardiac damage in Chagas disease, postulating that adipose tissue functions as a shield by abducting parasites and preventing parasites migration to the heart [21]. In contrast, the infection with *Plasmodium berghei* in mice with hypothalamic obesity accentuates damage to the host, which present severe brain damage, high parasitic loads, and an increase in pro-inflammatory cytokines such as IL-12 and IFN-γ [22]. In Malaria, caused by *Plasmodium falciparum*, obesity is considered as a risk factor in humans [23]. Our findings together show that in the case of infection by *T. pisiformis*, obesity is a factor that increases susceptibility, since obese-infected rabbits had a higher quantity of metacestodes. They also had a lower number of liver lesions attributable to changes in the physiology of the liver caused by obesity, since it has been proposed as an organ that mounts an immune response against the migration of the eggs of *T. pisiformis*, sequestering them through the formation of more granulomas and thus reducing the hatching and the number of metacestodes [24].

Total white and red cells count was not affected during the infection, however, eosinophilia was observed in the infected and heterophilia in the obese-infected rabbits. Our observations differ from a previous study where they reported that leukocytes increased from day 7 post infection until day 25 [5]. The differences may be related to the experimental conditions, since they used a different infective dose, observed different amounts of metacestodes lodged in the abdominal cavity of the rabbits, and also the sex of the rabbits was different. Eosinophilia has been reported in other parasitosis in rabbits; in case of infections with *Ascaris suum*, *Toxocara canis* and *Toxocaris leonina*, changes were observed in leukocytes, eosinophils and neutrophils, and the most marked eosinophilia was observed after 2 or 3 weeks after infection [25].

In the current work, infection by *T. pisiformis* eggs induced changes in lipid metabolism, since there was a decrease in serum cholesterol in both the infected and obese-infected groups. In obese an increase in triglycerides and VLDL was observed. These results agree with those reported for the infection with *S. mansoni*, a parasitosis that in mice causes a significant reduction in the serum lipid profile, and whose decrease was attributed to different metabolites that are synthesized by S. mansoni, arguing that they affect the liver tissue of the host [26]. In the case of cestodes, it was described that metacestodes of *T. solium* and *T. crassiceps* can synthesize androgens and estrogens through the transformation of certain precursors [27]. Lipids, particularly cholesterol and its metabolites, are required by some parasites [1]; this condition could explain the reduction of serum cholesterol levels in both obese and non-obese infected rabbits. Cholesterol reduction induced by parasitosis has been reported in humans. Natural infection with *S. haematobium* in obese individuals has a beneficial effect because it reduces the risk of developing cardiometabolic diseases [28]. Authors propose that the mechanism of the normalization of the lipid profile is due to the use of lipids in the synthesis of lipoproteins for the eggs of *S. haematobium*. This would also be a possibility in the infection with *T. pisiformis*.

Parasitic helminth infections usually induce immunosuppression. Cortisol is a hormone involved in the stress response, whose prolonged or excessive secretion can have physiological repercussions on the immune response and on reproductive functions [29,30]. The increase in serum cortisol in normal rabbits infected with *T. pisiformis* eggs, coincides with that previously observed in rabbits that were infected with *Eimeria coecicola* [31]; whereas in rabbits chronically infested with *P. cuniculi*, cortisol levels also tended to increase [32]. Furthermore, in obese patients, serum cortisol levels do not increase [33], coinciding with our observation in obese and obese-infected groups, where no increase in serum cortisol was observed. It is of interest to evaluate the function of the immune system and reproductive function in obese-infected rabbits.

In the current study it was observed that the liver weight of the rabbits was higher in the obese and in the obese-infected groups, this observation coincides with a previous work in which the high-fat diet and the increased weight in rabbits were associated with the infiltration of lipids into hepatocytes [12]. However, the increase in liver weight was proportional to the increase in body weight. The macroscopic evaluation performed on the livers showed 85% of the rabbits with fatty liver in the obese and obese-infected groups, whereas in the control and control-infected groups no changes were observed. The increase in visceral adipose tissue is associated with the triglycerides content of the liver [11], generating accumulation of lipids in the hepatocyte cytoplasm. This is associated with a progressive hepatic deterioration and with different degrees of liver fibrosis [34], supporting the increase in serum triglycerides and the increase in accumulated fatty tissue in obese rabbits.

Obese and infected rabbits with *T. pisiformis* eggs increased the weight of testicles, likewise in obese rabbits; it can be attributed to the fact that the parasite along with obesity could modify the host hormonal environment promoting the redistribution of adipose tissue to the testicles, since it has been described that the larval stage of *T. crassiceps* causes decreased testosterone and increased estradiol serum levels [35].

The infection with *T. pisiformis* caused a lower weight on the submandibular glands, both in normal weight (infected) and obese-infected groups. The submandibular glands in the rabbit are associated with sexual [36], territorial and hierarchical behavior of rabbits in both sexes [37,38]. The sexual and territorial function of the glands is dependent on the amount of serum testosterone in males [39], then, changes in submandibular glands could be attributed to modifications in serum testosterone levels. It was reported that rabbits induced to obesity and infected with *T. pisiformis* showed decreased chinning behavior, higher reaction time to mounts, and lower number of mounts [40]. These behaviors are highly associated with the level of serum testosterone.

In our study, suprascapular and visceral adipose tissue increased in obese-infected rabbits and peritesticular adipose tissue was increased in obese-infected rabbits. Studies in humans have shown that the distribution of adipose tissue are associated with risk to diseases. Excess of adipose tissue in the abdominal region, especially visceral adipose tissue, is associated with greater risks, although not all obese individuals develop the same pathologies or to the same degree of disease [41]. Our results partially coincide with those obtained in a study in rabbits fed a high fat diet in which an adipose tissue redistribution was observed, inducing central obesity and insulin resistance [42]. Although the etiology of obesity is multifactorial, it has been suggested that infection with specific pathogens can lead to an increase in adiposity [43,44]. In wild hares, it has been reported that the establishment of the larval stage of *Taenia pisiformis* presents a negative correlation with the body mass index, measured through the perirenal fat [45]. In this way, the changes observed in the distribution of adipose tissue in the different experimental circumstances could contribute to generate a more permissive environment for the development of the parasite, which involves changes in the immune and endocrine system.

## 4. Conclusions

Our findings indicate that the infection with *T. pisiformis* alters metabolic characteristics in the rabbit that can impact on the production and welfare of animals. These alterations are intensified in conditions of obesity. In future studies it will be important to clarify whether the infection with cestodes in obese individuals, has the ability to reverse the associated metabolic conditions such as metabolic syndrome and type II diabetes, and the relationship it maintains with the distribution of adipose tissue in the host, and not only to the amount of total fat. This work is a pioneer in characterizing the effects that infection by cestodes has on a natural host, in a bivalent scenario in which the obesity converges with the infection. It can be useful in veterinary clinical practice in which rabbits are diagnosed with obesity and there is also evidence in the clinical history of a probable infection with *T. pisiformis*. Furthermore, it can be useful as a study model in other parasitosis by cestodes such as *T. solium*, still prevalent in countries like Brazil, Mexico, and others. In addition, it opens the possibility of studying whether parasite antigens could contribute to the reduction of serum cholesterol.

## 5. Materials and Methods

### 5.1. Animals, Diets and Groups

Animal care and experimentation practices were followed with adherence to official Mexican regulations (NOM-062-ZOO-1999), which are in strict accordance with all applicable international, national, and institutional guidelines for the care and use of animals. Twenty-eight male New Zealand rabbits, 4.5 months old with an initial weight of 3.25 ± 0.12 kg were used; they were kept in individual cages (60 × 90 × 40 cm) under farm conditions, with an average annual temperature of 19 ± 3 °C. The 28 rabbits were assigned randomly into four groups of 7 rabbits each, and were fed according to the corresponding experimental treatment, providing them with water ad libitum. The control and the infected groups were fed with 180 gr of commercial maintenance pellets (Ganador^®^, Malta Cleyton, Mexico, minimum content 16% protein, 3% fat and 17% fiber) [46]. The obese and obese-infected groups were fed a high-fat diet (HFD) composed of commercial maintenance pellets added with 5% soybean oil and 5% pork fat. The HFD was offered *ad libitum* for 8 weeks previous to the infection [47], and thorough the experiment.

### 5.2. Infection

*T. pisiformis* infection was performed after 56 days of HFD; *T. pisiformis* proglottids collected from infected dogs, washed, identified and maintained according to the method described by Flatt and Moses (1975) were macerated in 5% saline in a mortar, and the viable eggs were quantified using a hemocytometer. The corresponding rabbits were inoculated with 3000 eggs via esophageal using a sterile plastic catheter [5].

### 5.3. Complete Blood Count, Liver Enzymes and Lipid Profile

At day 56 post infection, the rabbits were anesthetized with 35/5 mg/kg of ketamine/xylazine via IM [48]. A blood sample was obtained intracardiacally, separating a part of the whole blood in tubes with EDTA for the hematological analysis, which was performed with the Mindray BC-2800 analyzer equipment; another part of the blood was kept in tubes without anticoagulant, and serum obtained by centrifugation was used for the determination of cortisol, liver enzymes (alanine aminotransferase ALT and aspartate aminotransferase AST), and the lipid profile (triglycerides, cholesterol, high density lipoproteins HDL, very low density lipoproteins VLDL). The liver enzymes and lipid profile were determined using the specialized biochemical analysis equipment Roche COBAS C111, USA.

### 5.4. Serum Cortisol

Cortisol was measured in serum obtained individually at day 56 post infection and stored at −20 °C until analysis. Serum cortisol levels were determined in duplicate using a commercial radioimmunoassay kit (Pantex, Santa Mónica, CA) [32].

### 5.5. Humanitarian Sacrifice

The humanitarian sacrifice of the rabbits was performed at day 56 post-infection by administration of a lethal dose of sodium pentobarbital (100 mg/kg), on previously anesthetized rabbits with ketamine/xylazine (35/5 mg/kg) [48].

### 5.6. Parasite Load

Metacestodes of *T. pisiformis* were found in the thoracic and abdominal cavities, and the number of granulomatous lesions in the liver were recorded. The granulomas were observed in the capsule and the superficial hepatic parenchyma of the complete liver, the count was made double blind, considering a different granuloma each lesion surrounded by healthy tissue. The animals that presented granulomatous liver lesions or metacestodes were considered as infected [5].

### 5.7. Percentage of Tissue Weight

The liver, submandibular glands and testicles were dissected and weighed on an analytical balance [12], and the tissue percentage weight was obtained with respect to the total body weight of the rabbit. For the submandibular glands and testicles, the percentage was obtained by taking the sum of the weight of both glands.

### 5.8. Adipose Tissue

Visceral (distributed in epiploon and mesentery), perirenal (surrounding the kidney) suprascapular (found on the subcutaneous dorsal region between the two scapulae) and peritesticular (surrounding the testicle) adipose tissue were collected separately and weighed on an analytical balance [42]. The percentage of adipose tissue was calculated with respect to the body weight of each rabbit.

### 5.9. Statistical Analysis

The body weight was analyzed using a 2-way repeated measures mixed model approach (groups as the treatment factor and weeks as the repeated factor), followed by a multiple Tukey’s test. Number of metacestodes and hepatic granulomas were performed using a t test. The following parameters were analyzed using a one-way analysis of variance (ANOVA) followed by the Tukey’s multiple comparisons post hoc test to compare the responses between the different groups: weight gain, hemogram, hepatic enzymes, lipids, cortisol, liver, testicles and submandibular glands weight; percentage of liver, testicles and submandibular glands weight; total, suprascapular, visceral, peritesticular and perirenal adipose tissue. *P* values ≤ 0.05 were considered significant.

## Figures and Tables

**Table 1:** Complete blood count, liver enzymes and lipid profile of rabbits at day 56 after infection with *T. pisiformis* eggs.

Analyte	Control	Infected	Obese	Obese-Infected	Reference Values
RBC (× 10^12^)	6.4 ± 0.2	6.8 ± 0.2	6.6 ± 0.5	6.7 ± 0.3	6–6.8 [13]
WBC (× 10^9^)	6.9 ± 1.3	7.3 ± 1.1	6.3 ± 1.4	6.6 ± 0.9	6.8–9.7 [13]
Lymphocytes (%)	44.8 ± 2.5	43.3 ± 4.5	44.7 ± 9	38.2 ± 3.4	39–67 [13]
Eosinophils (%)	3.5 ± 0.2	4.3 ± 1.1 ^a^	2.3 ± 1	1.8 ± 1.1 ^b^	1–2 [13]
Neutrophils (%)	45.3 ± 2.4	45.7 ± 3.6 ^a^	46.7 ± 9.1 ^a^	55.5 ± 4.2 *^,b^	25–46 [13]
Basophils (%)	1.5 ± 1	1.7 ± 0.5	2.3 ± 1.4	1.3 ± 0.5	2.1–5 [13]
Monocytes (%)	4.8 ± 2.3	5 ± 1.3	4 ± 1.7	3.2 ± 2	1–8 [13]
MCH (Pg)	19.5 ± 0.6	19.6 ± 0.5	19.8 ± 0.8	19.5 ± 0.4	20–21 [13]
MCHC (g/dL)	30.5 ± 0.5	30.3 ± 0.5	30.2 ± 0.5	29.7 ± 0.2	32–34 [13]
AST(IU/L)	20.4 ± 5.6	16.4 ± 8.1	18.6 ± 7.8	19.2 ± 6.6	14–80 [14]
ALT(IU/L)	22.8 ± 3	20.9 ± 6.6	18.2 ± 4.8	20.6 ± 6.5	14–113 [14]
Cholesterol (mg/dL)	101.9 ± 4.5	88.3 ± 4.3 *^,a^	103.1 ± 3.8 ^b^	91.3 ± 2.2 *^,a^	10–80 [14]
Triglycerides (mg/dL)	93.6 ± 12.4	91.3 ± 11 ^a^	115.3 ± 9.9 *^,b^	81.3 ± 5.7 ^a^	15–160 [14]
HDL (mg/dL)	51.04 ± 4.3	47.2 ± 4.5	52.7 ± 3.5 ^a^	44.2 ± 2.6 ^b^	46–58 [14]
VLDL (mg/dL)	17.8 ± 2.57	19.3 ± 2.8 ^a^	25.8 ± 3 *^,b^	16.2 ± 1.1 ^a^	9–15 [14]

RBC, red blood cells; WBC, White blood cells; MCH, mean corpuscular hemoglobin; MCHC, mean corpuscular hemoglobin concentration; AST, Aspartate aminotransferase; ALT, Alanine aminotransferase. Values are expressed as mean ± SD. * Indicates differences between groups compared to control. Different letter indicates differences between treatments; *P* ≤ 0.05, ANOVA test, followed by aTukey’s post hoc test.

**Table 2:** Number of liver granulomas and metacestodes found at necropsy in rabbits infected with eggs of *T. pisiformis* after 56 days post-infection.

Rabbit	Granulomas		Metacestodes	
	Infected	Obese-Infected	Infected	Obese-Infected
1	1	0	5	4
2	6	0	2	13
3	3	0	7	22
4	2	1	10	7
5	0	0	5	21
6	10	0	7	18
7	3	3	5	7
Mean ± SD	3.6 ± 3.4	0.6 ± 1.1 *	5.8 ± 2.5	13.1 ± 7.3 *
Total	25	4	41	92

Values are expressed as mean ± SD. * Indicates differences between groups; *P* ≤ 0.05, T test.

**Table 3:** Changes in the localization of adipose tissue induced by infection.

Adipose Tissue (%)	Control	Infected	Obese	Obese-Infected
Perirenal	61 ± 5.2	69 ± 2.5 ^a^	56 ± 8.6 ^b^	60 ± 3.6
Suprascapular	17 ± 4.3	11 ± 1.7 *^,a^	23 ± 5.6 *	18 ± 1.0 ^b^
Visceral	16 ± 3.0	17 ± 2	18 ± 7.3	17 ± 3.4
Peritesticular	6 ± 2.4	3 ± 0.4 *	3 ± 0.5 *	5 ± 1.3

Values are expressed as mean ± SD. * Indicates differences of groups *versus* control. Different letter indicates differences between treatments; *P* ≤ 0.05, ANOVA test, followed by a Tukey’s post hoc test.
